# Electronic preoperative fasting abbreviation protocol: creation, application, and training of the patient care team

**DOI:** 10.1590/0100-6991e-20253755-en

**Published:** 2025-04-28

**Authors:** Rodrigo Costa Gonçalves, José Eduardo de Aguilar Nascimento, Marilia Arantes Rezio, Eula Cristina Machado Ferraz, Rachel de Carvalho

**Affiliations:** 1 - Hospital Estadual de Urgências Governador Otávio Lage de Siqueira (HUGOL) - Goiânia - GO - Brasil; 2 - Centro Universitário de Várzea Grande - Várzea Grande - MT - Brasil; 3 - Centro Estadual de Reabilitação e Readaptação Dr. Henrique Santillo (CRER) - Goiânia - GO - Brasil; 4 - Faculdade Israelita de Ciências da Saúde Albert Einstein - São Paulo - SP - Brasil

**Keywords:** Fasting, Clinical Protocols, Preoperative Care, Professional Training, Electronic Health Records, Jejum, Protocolos Clínicos, Cuidados Pré-Operatórios, Capacitação Profissional, Registros Eletrônicos de Saúde

## Abstract

**Introduction:**

The preoperative fasting time does not, in practice, meet current recommendations for preoperative care. The implementation of clinical protocols for shortening preoperative fasting has faced numerous barriers. The present study aims to evaluate whether the creation, application and professional training to use a fasting abbreviation protocol, linked to the electronic medical record, is capable of managing and reducing preoperative fasting time.

**Methods:**

The study was conducted in two public hospitals in Goiânia, Goiás, Brazil. The DMAIC project methodology (Problem Definition - Measurement - Analysis - Implementation and Control) was used. Initially, the preoperative fasting time was measured in both institutions and the possible root causes for its prolongation were analyzed. Based on this assessment, a fasting abbreviation protocol was developed, managed through the electronic medical record, and the preoperative fasting time was again measured. In parallel, training was carried out for the multidisciplinary team to apply the protocol.

**Results:**

Preoperative fasting time was high and superior to current recommendations in both hospitals. The causes for this prolongation were identified and treated. There was a reduction in preoperative fasting time in both institutions (11.50 vs 8.17 hours, p:0.000) and (8.77 vs 8.07 hours, p:0.025).

**Conclusion:**

The construction of a protocol, considering the needs of each institution, its management through electronic health records and the use of multiple methodologies for training patient care teams make it possible to reduce the duration of preoperative fasting.

## RESUMO

**Introdução:**

A duração do jejum pré-operatório não atende, na prática, as recomendações atuais de cuidados pré-operatórios. A implantação de protocolos clínicos de abreviação de jejum pré-operatório tem enfrentado inúmeras barreiras. O presente estudo tem como objetivo avaliar se a criação, aplicação e capacitação profissional para uso de um protocolo de abreviação de jejum, vinculado ao prontuário eletrônico, é capaz de gerenciar e reduzir o tempo de jejum pré-operatório.

**Métodos:**

O estudo foi conduzido em dois hospitais públicos de Goiânia, Goiás, Brasil. Foi utilizada a metodologia de projetos DMAIC (Definição do Problema - Medição - Análise - Implementação e Controle). Inicialmente foi medida a duração do jejum pré-operatório nas duas instituições e analisadas as possíveis causas raízes para seu prolongamento. Com base nessa avaliação, foi desenvolvido um protocolo de abreviação do jejum, gerenciado por meio do prontuário eletrônico e a duração do jejum pré-operatório foi novamente medida. Em paralelo, foi realizado o treinamento da equipe multiprofissional para aplicação do protocolo.

**Resultados:**

A duração de jejum pré-operatório era elevada e superior às recomendações atuais em ambos os hospitais. As causas para esse prolongamento foram identificadas e tratadas. Houve redução do tempo de jejum pré-operatório em ambas as instituições (11,50 vs 8,17 horas, p:0,000) e (8,77 vs 8,07 horas, p:0,025).

**Conclusão:**

A construção de um protocolo, contemplando as necessidades de cada instituição, seu gerenciamento por meio de registros eletrônicos de saúde e a utilização de múltiplas metodologias de capacitação de equipes de assistência ao paciente possibilitam a redução da duração do jejum pré-operatório.

## INTRODUCTION

Among the preoperative care, preoperative fasting aims to reduce the risk of regurgitation and bronchial aspiration in the anesthetic-surgical procedure^1^ ^-^ ^2^. Currently, shorter fasting times have been shown to be safe and improve clinical outcomes, feelings of thirst, hunger, and well-being in patients^3^ ^-^ ^6^. However, the reality preoperative fasting time around the world does not meet current recommendations^7^ ^-^ ^9^. A study conducted in 16 Brazilian hospitals indicated a median fasting time of 12 hours, which was even longer in hospitals using traditional fasting protocols^10^. 

The implementation of protocols aims to establish the best evidence-based routines in practice. Nonetheless, protocols for abbreviating operative fasting have been difficult to achieve in hospital services due to numerous barriers, including complex and unmanaged processes, decision-making based on hierarchy rather than protocol, absence of a multiprofessional work team, fragile communication channels, and problems in corporate education^11^ ^-^ ^13^.

We did not identify studies of implementation of this protocol using project methodology, electronic management, and professional training planning. In view of this, the present study aimed to verify whether the creation, application, and multiprofessional training for the use of a preoperative fasting abbreviation protocol, linked to the electronic medical record, would be able to manage and reduce the preoperative fasting time.

## METHODS

This is a study with a mixed design, with the initial stage of the observational/descriptive type and the implementation stage of the interventional protocol.

The methodology used to develop the protocol of this study was based on the DMAIC strategy (Problem Definition - Measurement - Analysis - Implementation and Control), of the Lean Six Sigma project methodology^14^.

We conducted the study in two public hospitals in the city of Goiânia, capital of the State of Goiás, Brazil.

The target population consisted of patients undergoing elective surgeries in both hospitals, whose sample size was defined by the pre- and post-intervention data collection period.

### Study design

To develop the protocol, we set up a multidisciplinary team to evaluate the fasting process, called the “Fasting Team”, involving members from the services of nutrition, nursing, surgery, anesthesiology, internal regulation, and information technology.

### Phase 1. Identification of surgical patients’ fasting times and determining factors

For six months, weekly meetings were scheduled with the “Fasting Team”, lasting one to two hours, to develop the project methodology and align the tasks, divided into five stages:

#### Step 1- Problem Definition

We presented the problem to the “Fasting Team”: difficulty in the abbreviation of preoperative fasting in institutions. We made a dialogical presentation of the existing literature review on the subject. We presented the data collection spreadsheet and conducted training on the attributions and format of data collection by the care teams involved in the surgical patient flow.

#### Step 2 - Measurement

We collected information on the fasting time of elective surgeries over a period of 30 days in a unit of the general surgical clinic of each of the participating institutions. We did evaluate the type of anesthesia used. The data collection spreadsheet was printed at the nursing station of the studied ward of each hospital. The orderlies responsible for the wards recorded the times referring to the last meal delivered to the patient before surgery, the ingestion of the shortening fasting solution (12.5% maltodextrin), when used, and the meal delivered after surgery. The nursing care team collected the other data from the spreadsheet, including the procedure starting time. In both services, fasting was initiated at the hospital and hospitalizations were performed at least on the day before surgery. The data on fast duration were presented in a meeting with the “Fasting Team”. 

#### Step 3 - Observational analysis

Through conversation circles with the “Fasting Team”, we mapped the flow of patients hospitalized with planned surgery, identifying the potential causes for the increase in fasting times at each stage of the flow, with the Brainstorm tool^15^. In each meeting, we exposed the stages of the flow and its barriers in the form of sticky notes (post-its) on a wall of the meeting room for better visualization by the team. In sequence, we grouped the causes using the Ishikawa’s Cause-Effect Diagram^16^. This diagram, also known as “fishbone”, has the purpose of organizing reasoning and hierarchically structuring the potential causes of a given problem or opportunity for improvement. It categorizes these root causes into six distinct groups of failures: method, material, workmanship, machinery, measurement, and environment. After this diagnostic stage, the project’s action plan was defined using the 5W2H methodology^17^. The 5W2H tool is a set of questions used to compose action plans quickly and efficiently. It consists of seven questions: What, Why, Where, Who, When, How, and How Much. 

### Phase 2. Development of the fasting abbreviation protocol

#### Step 4 - Implementation

The protocol was computerized in the institutions’ electronic medical record system (MV Soul) with the development of a protocol tab to manage the stages of the fasting protocol. We considered the information obtained in the analysis phase and identified as barriers to implementation, recording of times of meals received before and after surgery, prescription and bedside checking of shortening fasting solutions, and checklists of relevant information to ensure security in the protocol, in addition to the protocol closing document. The system captures the anesthetic induction start time for automated calculation of fasting times.

After this stage, we presented the training project for the care teams involved in the use of the computerized protocol. These teams were trained on how to fill out the protocol tabs and the new tasks to be performed, including those related to the education of patients and family members on preoperative fasting.

The planning of these training sessions involved the “Fasting Team”, through the evaluation of the barriers and facilitators identified in Phase 3. To that effect, we considered the importance of creating multiple training approaches, targeting different professional categories involved in the process, and the objective of transforming learning into performance enhancement, with improved fasting indicators and information to patients and communication among professionals^18^ ^,^ ^19^.

#### Step 5- Monitoring

In the monitoring phase, we collected automated data, provided via software for a new analysis of the preoperative fasting time. We developed indicators to monitor the various stages of the fasting protocol in the two institutions and to generate improvement cycles. 

### Statistical analysis

The dependent variable of the study was the preoperative fasting time, defined as the time elapsed between the last meal and the time of surgery, for patients without the use of a fasting abbreviation solution. For those using the solution, preoperative fasting time was defined as the time elapsed between the time of solution administration and the time of surgery.

We categorized the data into pre- and post-implementation of the computerized protocol in the two hospitals where the research took place. The pre-intervention quantitative data, collected during 30 days prior to the implementation of the protocol, were structured in double entry in the EpiData 3.1 software, with subsequent validation and export for analysis. We excluded from the statistical analysis data from patients whose times of surgery, times of the last meal, or times of fasting abbreviator ingestion were not recorded. We collected post-intervention data for two months, directly from the fasting time reports issued by the hospitals’ electronic medical records and imported them into the statistical analysis software.

We performed a descriptive analysis of the data, with calculation of the median, interquartile range, and variation of the preoperative fasting time, recorded in hours. Boxplot plots were constructed to evaluate data variability and dispersion. We assessed data distribution by the Kolmogorov-Smirnov test. We evaluated the difference between two medians (pre versus post implementation of the computerized protocol) by the Mann-Whitney test. Statistical analyses were performed using the Stata statistical package, version 12.0, considering statistical significance when p-value < 0.05.

### Ethical aspects

The research was approved by the Ethics in Research Committee of the Hospital Israelita Albert Einstein, according to resolution 466/2012 of the National Health Council, under opinion 5.836.799 (CAAE 65135722.5.0000.0071), together with the respective Committees of the co-participating institutions, under opinion 5.874.572 (CAAE 65135722.5.3002.0237).

## RESULTS

The median preoperative fasting time before the implementation of the protocol was 11.5 h (4.35 - 20.00) in hospital 1 and 8.77 h (2.83 - 16.65) in hospital 2. 

**Figure 1:**
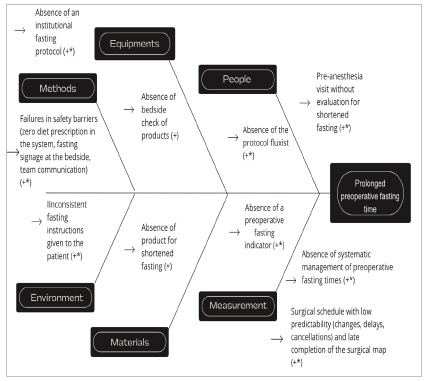
Determining factors for prolonging the preoperative fasting time. Hospital 1 factors (+) and hospital 2 factors (*).

The determining factors for prolonged fasting times were similar in both health units (Figure 1). However, hospital 2 already used a 12.5% maltodextrin solution for shortening fasting in an unmanaged form, which contributed to the shorter pre-intervention fasting time at that center. Action plans were drawn up to correct the determining factors. 

**Figure 2:**
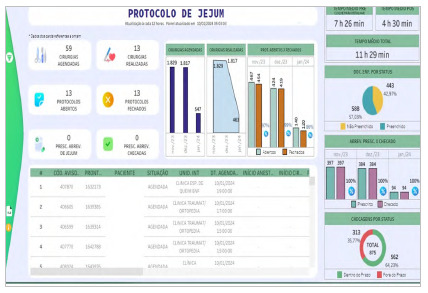
Panel for automated data collection and online management in the post-intervention period.

We developed a tab in the electronic medical record for managing patient information in the fasting abbreviation protocol, as well as a panel for online monitoring of data and indicators and automated collection of the duration of the preoperative fasting time after the intervention (Figure 2). The panel uses the Tableau Business Intelligence (BI) tool, accessing the database of the Hospital Management System “MV Sistemas” that operates with an Oracle database.

The protocol in both centers began with the evaluation of the surgical maps the night before the procedures, by a nutritionist of the protocol team. Each health institution was responsible for defining the time of the last solid meal and the ingestion of the shortening fasting solution, according to its surgical schedule, planning of the patients’ hospitalization time, and the delivery of meals. Therefore, not all surgical schedules were eligible for the shortening fasting solution. Patients with delayed gastric or esophageal emptying signaling at the preanesthetic visit were also not eligible for the use of this solution. 

Based on these definitions of the protocol, the nutritionist was responsible for prescribing the patient’s last solid meal and the shortening fasting solution in the electronic medical record, when indicated. The 12.5% maltodextrin solution was supervised by a nursing technician and its time was recorded by an electronic bedside checking system, scanning the barcodes of the patient’s wristband and the fasting abbreviation solution. The time of the last solid meal was recorded in a document with a structured field in the electronic medical record by the nutritionist. Patients included in the operative fasting protocol had prominent signs in the electronic medical record until the protocol was closed at the end of the fast. The nurses were responsible for signaling the fasting beds and emphasizing the time of surgery, the beginning of fasting, and the time of ingestion of the shortening fasting solution to patients and family members. 

The care teams were trained in both units to use the managed and computerized protocol. Some training sessions involved exclusively medical, nursing, or call center staff, while others involved the entire multi-professional team. Patients and family members were also contemplated with the development of a surgical booklet with valuable information. Different teaching methodologies were used: publication of a fasting protocol in the internal communication system, printed reminders posted on computers and displays, reminders on computer screens, workshops, gamification, corporate asynchronous distance learning (Distance Learning) classes, conversation circles with small groups at the workstations, and audits with individual feedback (Table 1).

**Table 1:** Corporate teaching strategies and teams involved.

TEACHING STRATEGIES	HOSPITAL 1	HOSPITAL 2
Fasting protocol available on the intranet	✔*	✔*
Printed reminders		✔#
Digital reminders	✔*	✔++
Printed educational booklet		✔+
Workshop/Seminars	✔**	
E-learning	✔#	
Small groups talks at workstations	✔*	✔*
Audits and individual feedbacks	✔*	✔*
Games	✔*	

*✔*: Strategy carried out * Multiprofessional team** Nursing team + Patients and family members ++ Call center # Medical team

Data analysis showed a reduction in the median preoperative fasting time at both institutions (Table 2).

**Table 2:** Preoperative fasting time before and after the intervention, in total hours and by data collection site.

	PRE-INTERVENTION		POST-INTERVENTION		
Health Unit	Median (IQR) (h)	Amplitude (h)	Median (IQR) (h)	Amplitude (h)	p-value*
Hospital 1	11.5 (9.00-15.58)	4.35-20.00	8.17 (6.27-9.42)	2.22-20.92	0.000
Hospital 2	8.77 (6.50-9.52)	2.83-16.65	8.07 (5.72-9.65)	2.05-19.92	0.025

h: hours; IQR: interquartile range (Q1-Q3); *Mann-Whitney test.

**Figure 3:**
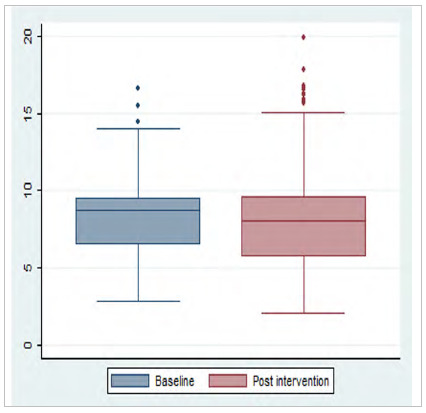
Boxplot of the preoperative (n=68) and post-intervention (n=603) fasting time of patients in hospital 1; p: 0.000.

There was a significant reduction in data variability in hospital 1, with 75% of the times below 9.42 h after the intervention (Figure 3).

**Figure 4:**
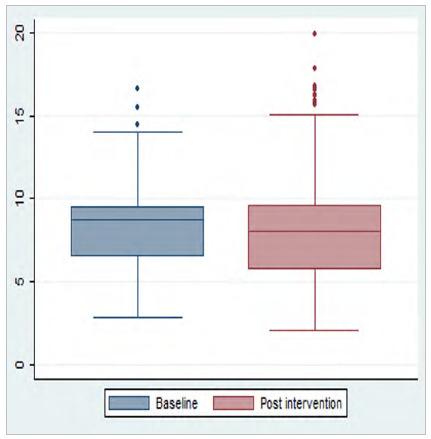
Boxplot of the preoperative (n=104) and post-intervention (n=1149) fasting time of patients in hospital 2; p: 0.025.

In hospital 2, despite the reduction in the median, 25% of the fasting times were longer than 9.65 h (Figure 4).

## DISCUSSION

The implementation of the computerized protocol for abbreviation of preoperative fasting and professional training allowed for a reduction in the duration of preoperative fasting in both health units studied. The construction of a protocol in an interdisciplinary way, adapted to the needs of each institution, its management in electronic format, and the use of multiple training methodologies are innovative techniques for the implementation of a preoperative fasting protocol.

As expected, the preoperative fasting times before the implementation of the protocol were prolonged in relation to current scientific recommendations and similar to those found in Brazilian and international studies^7^ ^-^ ^10^. The multicenter study of the Brazilian Group for the Study of Preoperative Fasting Time (BIGFAST) evaluated preoperative fasting in 3,715 patients in 16 Brazilian hospitals. The median fasting time was 12 hours, and fasting was significantly longer in hospitals using traditional fasting protocols (13 hours) than in hospitals adopting new guidelines (8 hours)10. We also observed this fact in hospital 2, which had a shorter initial fasting time because it already used a 12.5% maltodextrin solution, as recommended by guidelines^3^ ^,^ ^20^.

In the present study, the mapping of the process by the “Fasting Team” was fundamental for the identification of the main barriers to the protocol. The creation of a multidisciplinary team, including surgeons and anesthesiologists, for the development and implementation of the project has already been suggested by other studies, in which a collaborative approach adapted to local realities was a predictor of success in surgical care programs^11^ ^-^ ^13^. The use of project methodology techniques allows the involvement of the care team in the creation and development of the new workflow, ensuring greater learning and effectiveness of the processes^21^.

In contrast to what other studies have found^13^ ^,^ ^22^, anesthesiologists were not resistant to the protocol. We did not identify fear of bronchial aspiration or legal risks as barriers to the with the prescription of shortened fasting. In Brazil, the Federal Council of Medicine and the Brazilian Society of Anesthesiology validate this recommendation for anesthetic acts. The concern of anesthesiologists was that the lack of coordination between teams would lead to surgical cancellations. This complexity of processes, involving low predictability of appointments and surgical times, was observed in another study^11^ and was one of the main challenges of this project. The predictability of the surgical schedule in the two health units remained in need of improvement at the end of the study. We observed fasting times of more than 12 hours in situations such as postponement of the procedure, changes in the order of the surgical schedule, and cancellations made after the completion of the surgical map and planning of the fast.

The registration of contraindications to the use of shortening fasting solution during pre-anesthetic visits in wards (hospital 1) or outpatient clinics (hospital 2) ensured better communication with the nutrition service and greater safety in the process. Another study had already pointed out the benefits of the pre-anesthetic visit to the greater knowledge of patients about fasting recommendations^23^.

The “Fasting Teams” developed and published in both units a document supporting the protocol. However, other studies^11^ ^,^ ^24^ have identified that the existence of a protocol is not enough to improve results, minimize hierarchical decisions, and empower decision-making. The support of the hospitals’ clinical boards was crucial for the protocols to be treated as institutional. Improved communication channels, adequate monitoring, and feedback processes contribute to sustainability^11^.

Automated protocols can also promote greater adherence to the process and contribute to data management, directing actions towards numerous improvements^25^ ^,^ ^26^. Computerized data collection in the post-intervention allowed the management of the preoperative fasting duration of a larger number of patients when compared with paper-based management. Thus, the development of a computerized fasting protocol linked to the electronic medical record and a panel for managing fasting times and other protocol indicators, with filters by hospital areas, was innovative. This management was fundamental for the development of action plans and the construction of the project’s training.

The education of employees, patients, and family members facilitates the implementation of programs to accelerate surgical recovery^11^ ^,^ ^13^. In this way, we built a broad training matrix, involving all the teams involved in the process, using diversified methodologies, as planned by the “Fasting Team”. The combination of learning strategies is more effective for the success of improvement implementation^27^, and continuing education should involve the different professional categories that are part of the process. The creation of teams to build and monitor this implementation, as well as the use of electronic support systems for clinical decision-making, are recommended^18^ ^,^ ^28^. A systematic review produced by Cochrane evaluated implementation tools focused on health professionals, patients, and health organizations. It was not possible to identify the individual impact of each tool. However, printed educational materials, educational meetings, educational blitz, local opinion leaders, auditing and feedback, computerized reminders, and personalized interventions present evidence^28^. In addition, the outcome indicators are the most advanced level of evaluation of the effectiveness of training and show the real educational impact on health outcomes^29^.

### Study limitations

The methodology used allowed the identification of the existing barriers in both hospitals and individualized strategies to overcome them. However, other institutions can use the methodology, but build an implementation adapted to local needs.

Filling in data in the electronic medical record is the responsibility of the care teams and any failures cannot be eliminated.

The lack of an assertive surgical schedule did not allow the use of an abbreviating fasting solution at all surgical times, which could lead to better results.

It is not possible to separate the contribution of the project methodology from the computerization of the protocol and from the professional training in relation to the results of the present study.

The computerization of the protocol in MV Sistemas does not guarantee its implementation in other electronic medical records.

### Perspectives

It is desirable that future studies evaluate work overload and employee turnover as limiting factors for implementation and training and ways to overcome them in this protocol. Research involving strategies to optimize the surgical schedule, such as monitoring surgical times by related diagnosis groups and by surgical team and their impact on the predictability of fasting are also warranted.

## CONCLUSION

The elaboration and application of the protocol for abbreviation of preoperative fasting, contemplating the needs of each institution studied, its management in electronic format, and the use of multiple training methodologies for the multiprofessional team made it possible to reduce the preoperative fasting time in both health units. 
